# The calcium sensor, rather than the route of calcium entry, defines cerebellar plasticity pathways

**DOI:** 10.1073/pnas.2119598119

**Published:** 2022-02-22

**Authors:** Claire Piochon, Carole Levenes, Heather K. Titley, Christian Hansel

**Affiliations:** ^a^Professional Studies Department, University of Portsmouth, Portsmouth P01 2UP, United Kingdom;; ^b^Integrative Neuroscience and Cognition Center, Université de Paris, F-75006 Paris, France;; ^c^Edmonton Clinic Health Academy, University of Alberta, Edmonton, AB T6G 1C9, Canada;; ^d^Department of Neurobiology, University of Chicago, Chicago, IL 60637

Schonewille et al. (1) show that genetic deletion of GluN1 *N*-methyl-D-aspartate receptors (NMDARs) from cerebellar granule cells (GC-GluN1 ko), but not from Purkinje cells (PC-GluN1 ko), impairs long-term depression (LTD) at parallel fiber (PF) to PC synapses and vestibuloocular reflex (VOR) phase reversal. NMDARs are postsynaptically expressed at climbing fiber (CF) synapses (2). Challenging our findings that these postsynaptic receptors promote PF-LTD (3), the authors state “NMDARs in PCs are neither involved in PF-PC synaptic plasticity nor required for cerebellar motor learning” (1). We respectfully reject this conclusion.

For LTD, an older-generation protocol was used, in which PF bursts are paired with 400-Hz CF bursts (1). We recently assessed LTD outcomes when recordings are performed in a physiological ionic milieu ([Ca^2+^]_o_ = 1.2 mM; [Mg^2+^]_o_ = 1 mM; ref. 4) and observed that LTD does not result from PF burst pairing with single complex spikes, even when 400-Hz CF stimulation is used (5). Instead, LTD requires complex spike firing in naturally occurring clusters, a prolonged pattern favoring activation of the critical calcium sensor CaMKII (5–7). It remains to be determined whether or not LTD observed under these realistic conditions depends on postsynaptic NMDARs.

**Fig. 1.**
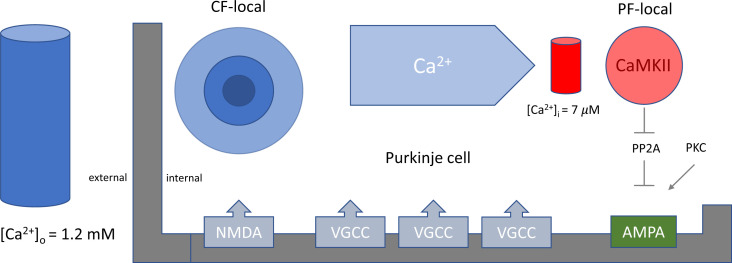
Postsynaptic NMDA receptors promote cerebellar LTD.

Nonrealistic protocols remain informative about mechanistic plasticity aspects. For example, our finding that postsynaptic NMDAR blockade prevents LTD shows that NMDAR activation principally can initiate LTD (3). The physical distance between CF and PF synapses necessitates the coupling of the CF-local calcium event (e.g., NMDA spike) to a PF-local one, likely by activation of voltage-gated calcium channels (VGCCs) (Fig. 1). A consequence is that direct VGCC activation bypasses NMDARs. We believe that this is the effect seen in ref. 1. A similar debate took place >30 y ago regarding NMDARs in hippocampal long-term potentiation (LTP). While NMDARs were known to promote LTP (8), it was subsequently demonstrated that conditions favoring VGCC activation induce LTP by bypassing NMDARs (9). The conclusion was not that NMDA receptors are not important for LTP. Rather, it was understood that the calcium sensor initiates LTP as long as appropriate calcium trigger signals are provided.

Finally, it is unclear why the authors chose VOR adaptation as a motor learning test (1), which, in their view, does not involve LTD but involves LTP (10). This is an inappropriate selection to assess the role of NMDARs in LTD. It is notable that the VOR gain increase observed on day 4—which, in the classic view, results from LTD—is significantly impaired in PC-GluN1 ko mice. This observation supports an interpretation contrary to that offered by the authors: Postsynaptic NMDAR-dependent LTD mediates aspects of VOR adaptation, and possibly further (untested) motor learning phenomena.
